# Triflumizole as a Novel Lead Compound for Strigolactone Biosynthesis Inhibitor

**DOI:** 10.3390/molecules25235525

**Published:** 2020-11-25

**Authors:** Kojiro Kawada, Yuya Uchida, Ikuo Takahashi, Takahito Nomura, Yasuyuki Sasaki, Tadao Asami, Shunsuke Yajima, Shinsaku Ito

**Affiliations:** 1 Department of Bioscience, Tokyo University of Agriculture, Setagaya, Tokyo 156-8502, Japan; kawa7440@gmail.com (K.K.); uhi-yu1998@ezweb.ne.jp (Y.U.); y1sasaki@nodai.ac.jp (Y.S.); yshun@nodai.ac.jp (S.Y.); 2 Graduate School of Agricultural and Life Sciences, The University of Tokyo, Bunkyo, Tokyo 113-8657, Japan; takahashi.190@gmail.com (I.T.); asami@g.ecc.u-tokyo.ac.jp (T.A.); 3 Center for Bioscience Research and Education, Utsunomiya University, Utsunomiya, Tochigi 321-8505, Japan; tnomura@cc.utsunomiya-u.ac.jp

**Keywords:** strigolactone, P450 inhibitor, screening, triflumizole, rice

## Abstract

Strigolactones (SLs) are carotenoid-derived plant hormones involved in the development of various plants. SLs also stimulate seed germination of the root parasitic plants, *Striga* spp. and *Orobanche* spp., which reduce crop yield. Therefore, regulating SL biosynthesis may lessen the damage of root parasitic plants. Biosynthetic inhibitors effectively control biological processes by targeted regulation of biologically active compounds. In addition, biosynthetic inhibitors regulate endogenous levels in developmental stage- and tissue-specific manners. To date, although some chemicals have been found as SL biosynthesis inhibitor, these are derived from only three lead chemicals. In this study, to find a novel lead chemical for SL biosynthesis inhibitor, 27 nitrogen-containing heterocyclic derivatives were screened for inhibition of SL biosynthesis. Triflumizole most effectively reduced the levels of rice SL, 4-deoxyorobanchol (4DO), in root exudates. In addition, triflumizole inhibited endogenous 4DO biosynthesis in rice roots by inhibiting the enzymatic activity of Os900, a rice enzyme that converts the SL intermediate carlactone to 4DO. A *Striga* germination assay revealed that triflumizole-treated rice displayed a reduced level of germination stimulation for *Striga*. These results identify triflumizole as a novel lead compound for inhibition of SL biosynthesis.

## 1. Introduction

Strigolactones (SLs) are a group of terpenoid lactones derived from carotenoids. More than 20 SLs, including 4-deoxyorobanchol (4DO), orobanchol, and methyl carlactonoate (MeCLA), have been reported from various plant species [1]. SLs function as plant hormones that regulate several important developmental processes in plants, including the regulation of shoot branching and stress tolerance [2,3,4]. In addition, SLs are allelochemicals that induce branching of hyphae in arbuscular mycorrhizal fungi and germination in root parasitic plants [5,6].

Root parasitic plants, such as *Orobanche* spp. and *Striga* spp., are harmful for crop production in Sub-Saharan Africa, the Middle East, and Asia [7]. In the savanna regions of Africa alone, annual losses amount to $US 7 billion [8]. Host plant mutants deficient in SL biosynthesis prevent infection by root parasitic plants. Thus, it is possible that inhibition of SL biosynthesis could reduce these damages [3].

Inhibitors of the biosynthesis of bioactive substances are useful to regulate biosynthetic pathways. While loss-of-function mutants in a biosynthetic pathway may result in a lethal phenotype, biosynthesis inhibitors can control the endogenous levels of metabolites in a developmental stage- and tissue-specific manner [9]. In addition, although gene knockout in multiple paralogous genes rarely produces a clear phenotype, biosynthetic inhibitors can overcome such gene redundancy in many cases. Therefore, the use of biosynthetic inhibitors is a valuable way to evaluate the physiological roles of endogenous substances.

**Figure 1:**
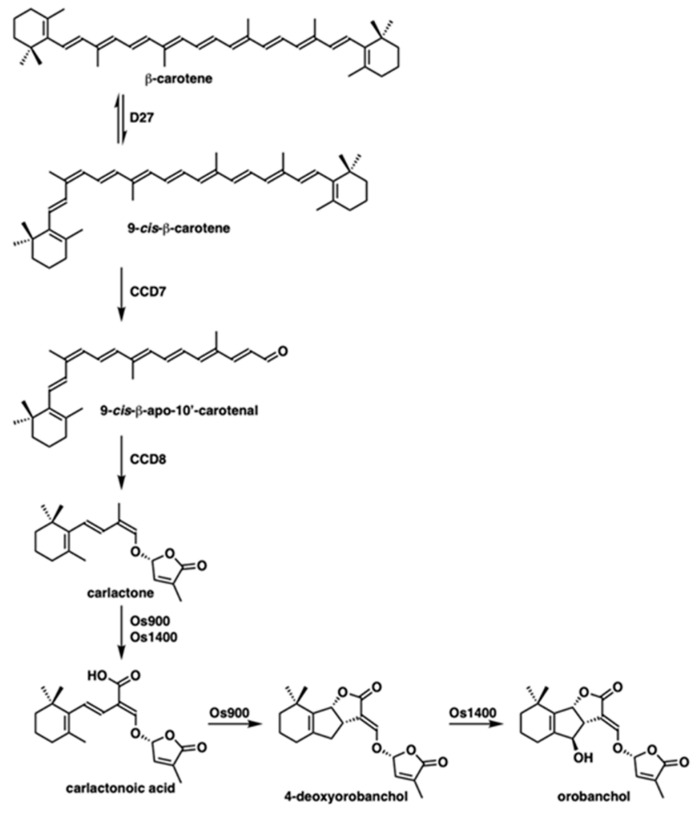
Strigolactone biosynthesis pathway in rice.

Genetic analysis using a series of branching mutants has revealed several enzymes involved in SL biosynthesis. In the first step, all-*trans*-β-carotene is converted to 9-*cis*-β-carotene by the D27 carotenoid isomerase [10]. Carlactone (CL), which is synthesized from 9-*cis*-β-carotene by two carotenoid cleavage dioxygenases, CCD7 (AtMAX3 in *Arabidopsis*/D17 in rice) and CCD8 (AtMAX4 in *Arabidopsis*/D10 in rice), is an important precursor of SLs [10,11]. In rice, cytochrome P450 proteins (CYPs) catalyze the conversion of CL to SLs. Os900 (CYP711A2) oxidizes CL to 4DO via carlactonoic acid (CLA). 4DO is converted to orobanchol by Os1400 (CYP711A3). Os1400 also catalyzes the conversion of CL to CLA (Figure 1). In *Arabidopsis*, AtMAX1 (CYP711A1) only shows catalytic activity from CL to CLA [12,13].

It has been reported that nitrogen-containing heterocyclic compounds, including imidazole and triazole groups, have inhibitory activities against various CYPs in mammals, microorganisms, and plants [14,15,16,17,18]. Thus, these compounds are good candidates as SL biosynthesis inhibitors. In our previous study, we screened for chemicals that induce second tiller bud outgrowth in rice, which is characteristic of SL-deficient mutants. The triazole-containing compound TIS13 was identified as the lead compound for SL biosynthesis inhibitors [19]. A structure-activity relationship study based on the structure of TIS13 identified two potent and specific SL biosynthesis inhibitors, TIS108 and KK5 [20,21]. In addition to TIS13, of seven azole derivatives identified as fungicides, tebconazole was implicated a lead compound for SL biosynthesis inhibitors [22]. Furthermore, though CYPs are not the target proteins, hydroxamic acid derivatives have been also reported as SL biosynthesis inhibitor [23]. However, the studies of SL biosynthesis inhibitor are still underdeveloped. Until now, only three groups of chemicals (TIS13, tebuconazole, and hydroxamic acid derivatives) have been found to be SL biosynthesis inhibitors (Supplementary Material Figure S1).

In this study, to find a novel compound which has inhibitory activity against SL biosynthesis, we evaluated the effects of several heterocyclic nitrogen-containing compounds on SL biosynthesis. The results showed that triflumizole is a novel lead compound for SL biosynthesis inhibition in rice.

## 2. Results

### 2.1. Screening for Compounds that Decrease 4DO Levels in Rice Root Exudates

**Figure 2:**
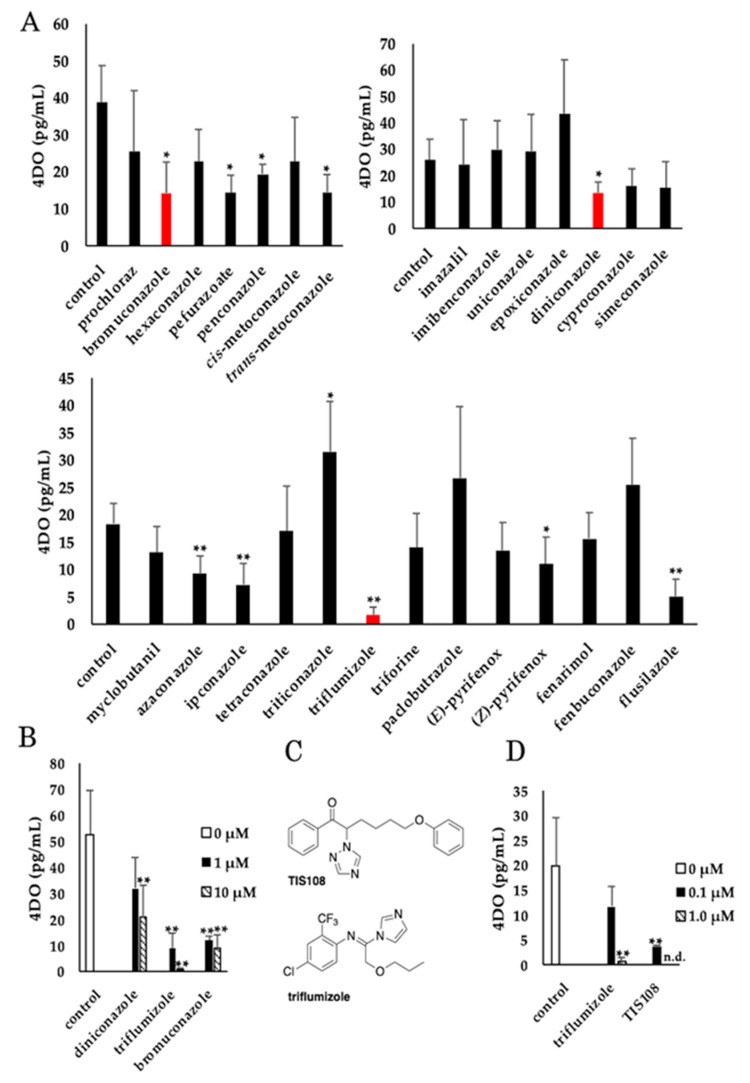
Effects of tested compounds on 4DO levels in rice root exudates. The levels of 4DO in rice root exudates treated with (**A**) 1 μM and (**B**) 1 or 10 μM of the screened compounds determined by LC-MS/MS. (**A**) Red bars represent the compounds which showed the lowest 4DO level in each experiment. (**B**) White, black, and slash bars are represented as the application of the 0, 1, and 10 μM compounds, respectively. (**C**) The chemical structures of TIS108 and triflumizole. (**D**) Comparison of the effect of 0.1 and 1 μM TIS108 and triflumizole on 4DO levels in the exudate of rice roots. White, black, and slash bars are represented as the application of the 0, 0.1, and 1 μM compounds, respectively. n.d.: not detected. The data are mean ± SD (*n* = 3 in panels A and B, *n* = 5 in panel D). * and ** denote statistically significant difference from the level in control plants (*t* test; *p* < 0.05 and *p* < 0.01, respectively).

To screen for compounds that inhibit SL biosynthesis in rice seedlings, the effects of 27 P450 inhibitors were evaluated, which are commercially available, as they are used as agrochemicals (Supplementary Material Figure S2). The inhibition of SL biosynthesis was determined by measuring the level of 4DO, a major endogenous SL in rice, in rice root exudates by liquid chromatography-tandem mass spectrometry (LC-MS/MS). It is known that the endogenous level of 4DO in roots correlates with that in root exudates [20]. To allow easy detection of 4DO by LC-MS/MS, rice seedlings were grown under phosphate-deficient conditions [3,24]. First, the effect of the tested compounds at 1 μM on 4DO levels were measured in root exudates. In comparison with the 4DO levels in root exudates of untreated rice, 11 compounds displayed statistically significant differences in their 4DO levels (Figure 2A). Diniconazole, triflumizole, and bromuconazole showed comparatively good 4DO inhibitory activity among the tested compounds (Figure 2A) and were selected for further analysis. Next, the reduction of 4DO levels in root exudates by the three compounds was evaluated at the concentrations of 1 and 10 μM. All three reduced the levels of 4DO in a dose-dependent manner. Of the three compounds, triflumizole showed the strongest 4DO inhibitory activity in root exudates (Figure 2B). Triflumizole is a fungicide that acts by inhibiting P450, which catalyzes ergosterol biosynthesis (Figure 2C) [25]. This is the first observation in which triflumizole inhibits SL production. Furthermore, the structure of triflumizole differs markedly from those of other known SL biosynthesis inhibitors. For instance, TIS108 has ketone and triazole moieties, while triflumizole has anil (*N*-phenyl imine) and imidazole moieties (Figure 2C). The inhibition of 4DO production in rice root exudates by triflumizole and TIS108, both of which are potent SL biosynthesis inhibitors, was compared (Figure 2D). Rice treated with 1.0 µM triflumizole or TIS108 displayed significantly reduced 4DO levels. Although the 4DO levels in 0.1 µM triflumizole-treated rice were higher than those in 0.1 μM TIS108-treated rice, the reduction rate of the 4DO levels and the novel chemical structure of triflumizole indicate that the compound could be a good lead compound for development of a potent SL biosynthesis inhibitor.

### 2.2. Os900 Inhibition Assay of Tested Compounds

**Figure 3:**
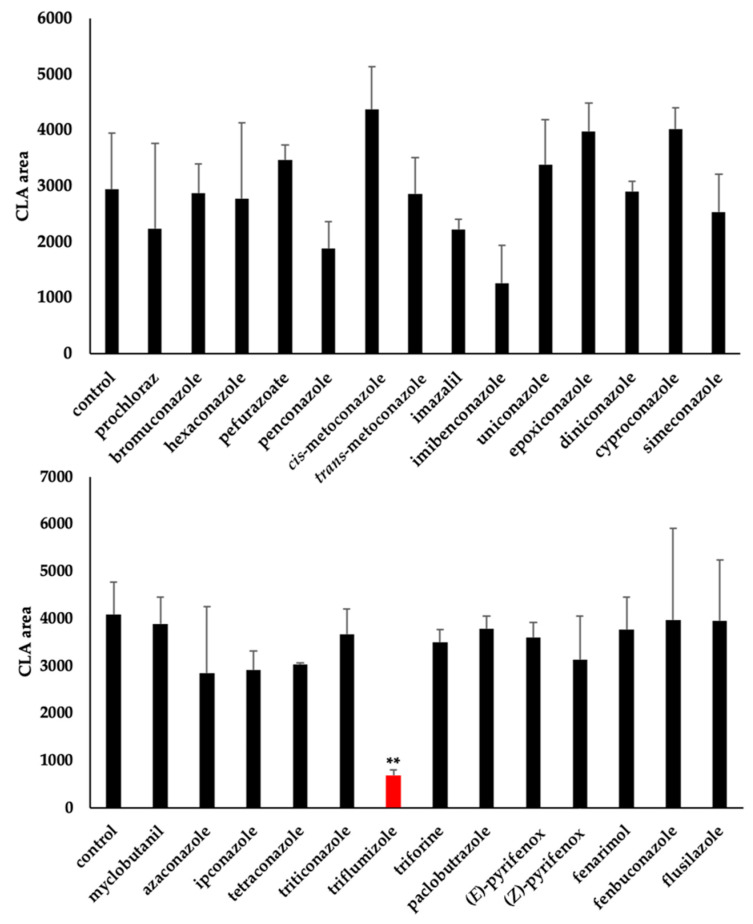
Effect of screening compounds on Os900 in vitro. CLA levels following the addition of 10 μM of each compound to microsomal proteins, including Os900. CLA levels were determined by LC-MS/MS. Red bar represents the compound which showed the lowest CLA area. The data are presented as mean ± SD (*n* = 3). ** denotes statistically significant difference from the CLA level in the no-application of chemicals (control) (*t* test; *p* < 0.01).

To determine the effect of the tested compounds at 10 μM on Os900, which is a P450 protein in the SL biosynthesis pathway, an Os900 inhibition assay was performed. Since Os900 is involved in the two-step conversion from CL to CLA and CLA to 4DO [13], we used CL as a substrate and measured CLA levels by LC-MS/MS. As reported by Yoneyama et al. [13], microsomal proteins, including Os900 from heterologously expressed yeast were collected and used for the assay. The addition of triflumizole to Os900-containing microsomal proteins produced a significant reduction in CLA levels (Figure 3). Diniconazole and bromuconazole did not affect the CLA levels in the Os900 inhibition assay, although these compounds reduced the 4DO levels in root exudates.

### 2.3. Effect of Triflumizole on SL Biosynthesis

Of the test compounds, triflumizole treatment reduced 4DO levels most effectively in rice root exudates. In addition, triflumizole inhibited Os900 activity in vitro. Therefore, we selected triflumizole as a candidate for a novel lead compound of an SL biosynthesis inhibitor in rice and performed further experiments.

**Figure 4:**
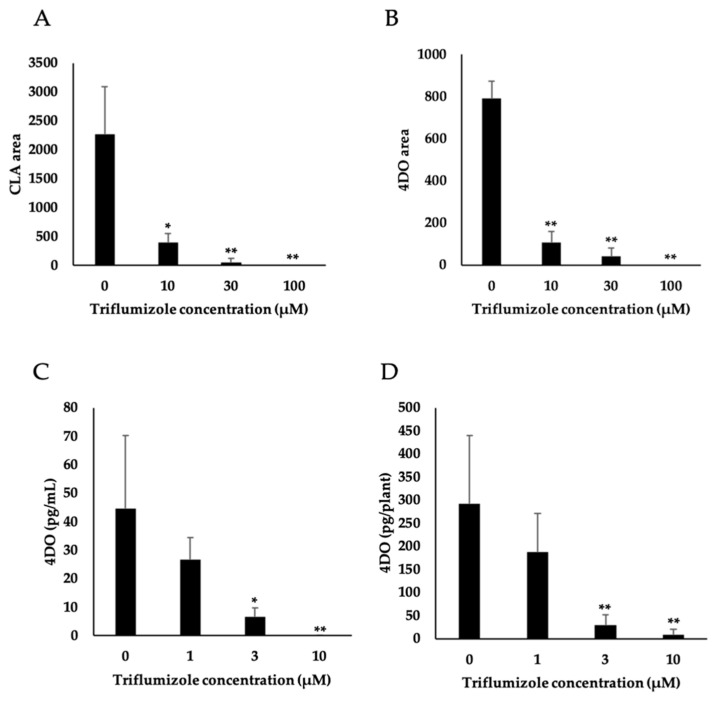
Effect of triflumizole on rice SL biosynthesis. Effect of triflumizole addition to Os900 expressed yeast microsomal protein on the levels of CLA (**A**) and 4DO (**B**) as determined by LC-MS/MS. The data are mean ± SD (*n* = 3). Statistical difference from the CLA (**A**) or 4DO (**B**) level of the 0 μM triflumizole is indicated by * (*t* test; 0.01 < *p* < 0.05) and ** (t test; *p* < 0.01). Effect of triflumizole addition to rice on 4DO levels in rice root exudates (**C**) and roots (**D**). The data are means ± SD (*n* = 5). Statistically different from the 4DO level of the control plants ((*) = *t* test; 0.01 < *p* < 0.05, (**) = *t* test; *p* < 0.01).

To confirm that triflumizole regulates SL biosynthesis by inhibiting Os900 activity in vitro, the Os900 inhibition assay was performed using various concentrations of triflumizole. The conversion of CL to CLA and CL to 4DO was determined (Figure 4A,B). The addition of 10–100 μM triflumizole to Os900-containing microsomal proteins reduced the levels of both products in a dose-dependent manner (Figure 4A,B). This pattern of reduction indicated that one of the target enzymes of triflumizole could be Os900. To confirm that triflumizole actually inhibits SL biosynthesis in vivo, we analyzed the endogenous levels of 4DO in rice roots and root exudates (Figure 4C,D). The 4DO levels in both root exudates of rice treated with triflumizole were reduced in a dose-dependent manner at concentrations ranging from 1–10 μM. These results suggested that triflumizole inhibits SL biosynthesis in vitro and in vivo.

### 2.4. Side Effect of Triflumizole

**Figure 5:**
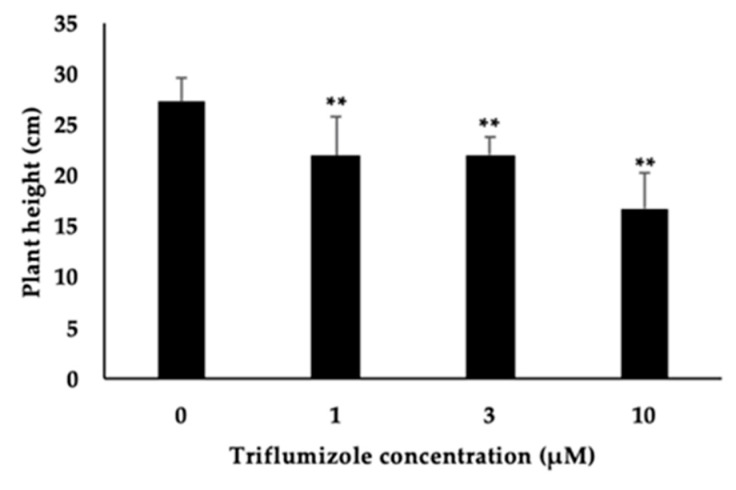
Plant height of 23-day-old rice seedling supplemented with 1, 3, or 10 μM triflumizole for two weeks. The data are expressed as mean ± SD (*n* = 10). ** denotes statistically significant difference from the plant height of non-treated triflumizole (*t* test; *p* < 0.01).

P450 inhibitors containing triazole and imidazole groups often induce dwarfism in rice because of the presence of various types of P450s in the gibberellin and brassinosteroid biosynthesis pathways. TIS13 induces a severe dwarf phenotype to rice as a side effect, which is rescued by the addition of gibberellin to rice [19]. Triadimefon is a fungicidal triazole-type P450 inhibitor but inhibits gibberellin and brassinosteroid biosynthesis [26]. Based on these results, we examined the side effects of triflumizole by measuring the height of rice plants from the ground to the highest leaf treated with triflumizole. Triflumizole treatment slightly but significantly reduced plant height in a dose-dependent manner (Figure 5). TIS108 and KK5 do not reduce the height of rice plants when applied at 50 μM [21]. The collective findings indicate that triflumizole seems to inhibit the enzymatic activity of Os900 as well as the activities of other P450 proteins in the gibberellin and/or brassinosteroid biosynthesis pathways.

### 2.5. Striga Germination Assay

**Figure 6:**
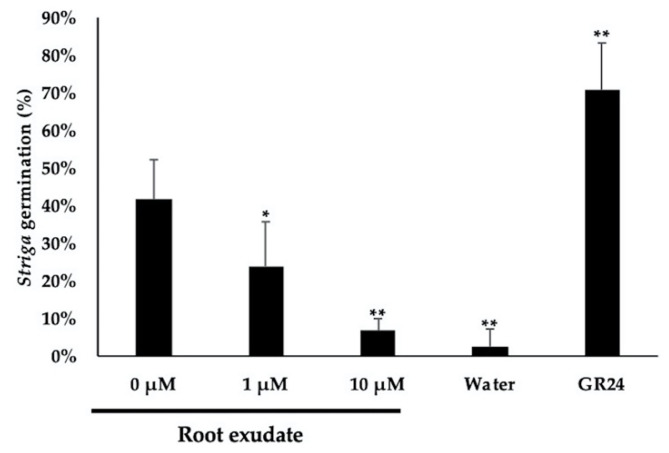
Germination of *Striga* seeds in the culture media of root exudates from untreated rice (0 μM triflumizole) and rice treated with 1 and 10 μM triflumizole. GR24 (synthetic SL analog) and sterile water were used as positive and negative controls, respectively. The data are expressed as mean ± SD of five samples. Statistically different from the germination percentage of 0 μM triflumizole ((*) = *t* test; 0.01 < *p* < 0.05, (**) = *t* test; *p* < 0.01).

Root parasitic plants, such as *Striga* and *Orobanche*, sense SLs, which induces germination. Hence, the lower levels of SLs in triflumizole-treated rice could reduce *Striga* germination. We checked the effects of treating rice seedlings with triflumizole on *S. hermonthica* germination. The root exudates of triflumizole-treated rice significantly reduced *Striga* germination in a dose-dependent manner (Figure 6). In addition, the co-application of triflumizole with GR24 (synthetic SL analog) induced *Striga* germination (Supplementary Material Figure S3), suggesting that the reduction of *Striga* germination treated with the root exudates of triflumizole-treated rice is not caused by the direct inhibition of *Striga* germination. These results corresponded with the results of the 4DO analysis in root exudates and roots, and indicated that triflumizole could control the germination of root parasitic plants dependent on SLs as a germination trigger.

## 3. Discussion

To identify novel lead compounds as SL biosynthesis inhibitors, we evaluated 27 P450 inhibitors known to be fungicides and plant growth retardants. Three compounds significantly reduced the 4DO levels in the root exudates of rice when applied at 1 μM. In particular, triflumizole reduced the levels of 4DO in rice roots and root exudates at concentrations ranging from 0.1–10 μM. Hydroxamic acid and TIS108 derivatives have been identified as inhibitors of SL biosynthesis. However, the activity of anil compounds, such as triflumizole, has not been reported. We observed that triflumizole treatment inhibited the Os900-mediated of CL to CLA and 4DO. These results indicate that Os900 is a target protein of triflumizole and that the inhibition of Os900 activity by triflumizole leads to the reduction of 4DO levels in roots and root exudates of rice. However, it is unclear whether triflumizole inhibits the activity of other CYP711As, including Os1400 and AtMAX1. To know the target enzymes of triflumizole in detail, we intend to assess the effects of triflumizole on other CYP711As by the inhibition assay of those enzymes in the near future. Interestingly, treatment with 10 µM bromuconazole and diniconazole reduced 4DO levels in rice exudates by half of the levels in control rice, although these compounds did not inhibit the enzymatic activity of Os900. The D27 and CCD families consist of iron-containing proteins [27,28,29,30]. Iron ions at the active site of CCD proteins are chelated by four histidine residues [31]. It is possible that the heterocyclic nitrogen-containing compounds, such as imidazole and triazole, chelate iron and inhibit CCD proteins. Considering the collective results, it is plausible that bromuconazole and diniconazole inhibit SL biosynthesis by inhibiting the activities of the D27, CCD7, or CCD8 enzymes involved in SL biosynthesis. Plants harbor various P450s involved in plant hormone biosynthesis and metabolism. These hormones include gibberellin, brassinosteroid, and abscisic acid [32,33,34,35], suggesting the possibility that triflumizole inhibits these P450s. Gibberellin- or brassinosteroid-deficient mutants cause dwarfism in rice and display the dwarf phenotype. Paclobutrazol, which is a gibberellin biosynthesis inhibitor, produces the gibberellin deficient mutant-like phenotype [36,37,38]. It was observed that 23-day-old rice plants treated with triflumizole for 2 weeks showed the dwarf phenotype, suggesting that triflumizole inhibits the CYP711A family as well as other P450s, including those involved in gibberellin and brassinosteroid biosynthesis.

In this study, it was found that triflumizole inhibits SL biosynthesis in rice by inhibiting Os900 activity. As SLs play an important role in plant development and resistance of environmental stress [1,39], triflumizole may be useful tool for analyzing the influence of SL biosynthesis inhibition and controlling the damage of root parasitic plants. However, because the dwarf phenotype is a side effect of triflumizole, a structure-activity relationship study using triflumizole as the lead compound will be necessary in the near future to attempt to reduce this side effect and conclusively establish triflumizole derivatives as a specific SL biosynthesis inhibitor.

## 4. Materials and Methods

### 4.1. Rice Growth Condition

The Nipponbare normal species was used as the wild-type rice. Rice seeds were grown as described in a previous study [3]. Rice seeds were sterilized by 2.5% sodium hypochlorite, added to sterile water, and incubated at 25 °C in the dark for 2 d. The germinated seeds were transferred into a phosphate-deficient hydroponic culture medium adjusted to pH 5.7 (1 mM NH_4_NO_3_, 0.3 mM K_2_CO_3_, 0.4 mM MgCl_2_, 0.2 mM CaCl_2_, 45 μM Fe-EDTA, 50 μM H_3_BO_3_, 9 μM MnSO_4_, 0.3 μM CuSO_4_, 0.7 μM ZnSO_4_, and 0.1 μM Na_2_MoO_4_), solidified with 0.7% agar, and cultivated at 25 °C under fluorescent white light with a 14-h light and 10-h dark photoperiod for 7 d. Each seedling was transferred to a brown vial with 12 mL of phosphate-deficient medium and incubated under the same conditions for 6 d. After that, these seedlings were transferred to a new brown vial containing 12 mL of the same media supplemented with the tested compounds and grown under the same conditions for 1 d. All tested compounds were purchased from FUJIFILM Wako Pure Chemical Corporation (Osaka, Japan). To measure the 4DO levels and the *Striga* germination percentage, hydroponic culture media and roots were collected. 4DO levels were measured in three (Figure 2A,B) or five (Figure 2D and Figure 4C,D) biological replicates. To assess plant growth, the germinated seeds were transferred into a hydroponic culture medium, containing 0.6 mM Na_2_HPO_4_ solidified with 0.7% agar and cultivated at the same condition as previously described [3]. 9-day-old seedings were transferred to a new brown vial containing 12 mL of a hydroponic culture media supplemented with triflumizole for two weeks. Plant height was measured from the ground to the highest leaf of the plant in 10 biological replicates that were 23 days old [40].

### 4.2. Quantification of the Endogenous 4DO Level

To analyze 4DO in rice root exudates, the hydroponic culture medium was extracted with ethyl acetate twice after the addition of deuterium-labeled 5-deoxystrigol (d_6_-5DS; 400 pg) as an internal standard [41]. The organic layer was concentrated in vacuo. To analyze 4DO in roots, roots were homogenized in acetone with d_6_-5DS added and filtered the suspension. The filtrates were concentrated and dissolved in 10% acetone. The solution was loaded onto Oasis HLB 3-mL cartridges (Waters, Milford, MA, USA), washed twice with deionized water (3 mL), eluted twice with acetone (3 mL), concentrated, and dissolved in 1 mL of *n*-hexane:ethyl acetate (85:15). The solution was loaded on a Sep-Pak silica 1-mL cartridge (Waters), flowed twice with 1 mL of *n*-hexane:ethyl acetate (85:15), eluted three times with *n*-hexane:ethyl acetate (65:35), and concentrated in vacuo [42].

The concentrates were dissolved in acetonitrile:deionized water (1:1) and subjected to LC-MS/MS analysis performed as reported previously [42].

### 4.3. Examination of Inhibition of Os900

#### 4.3.1. Conditions of the Enzyme Assay

Heterologous expression of Os900, collection of microsomal proteins, and the enzymatic reaction were performed as described in a previous study [13]. *Rac-CL* was synthesized according to a previous study [43].

*Rac*-CL and Os900 were incubated with NADPH (50 μM) in the presence or absence of test compounds at 28 °C for 40 min. The reaction mixture was quenched with 100 μL of ethyl acetate and added 50 μL of water. The mixture was extracted with 100 μL of ethyl acetate three times. The combined ethyl acetate fractions were concentrated in vacuo. The concentrates were dissolved in acetonitrile and analyzed by LC-MS/MS.

#### 4.3.2. LC-MS/MS Analysis of CLA Level

LC-MS/MS analysis was performed with a quadruple/time-of-flight tandem mass spectrometer (TripleTOF5600 system; SCIEX, Framingham, MA, USA) and an ultrahigh-performance liquid chromatograph (Nexera; Shimadzu, Kyoto, Japan) provided with a reversed-phase column (Acquity UPLC BEH-C18, 2.1 × 50 mm, 1.7 µm; Waters).

In the separation by ultrahigh-performance liquid chromatography, we used water (solvent A) and acetonitrile (solvent B), which both contained 0.1% (*v*/*v*) formic acid. The mobile phase had a flow rate of 0.2 mL/min and was altered with linear gradient of 20 to 60% solvent B (0–2 min), 60 to 100% solvent B (2–9 min), and 100 to 20% solvent B (12–12.10 min). The parent ions (*m*/*z*) were 331.2 for CLA. The samples were quantified using fragment ions 113.1 for CLA in the negative mode.

### 4.4. Striga Assay

The *Striga* germination assay was performed as previously described [44]. *Striga hermonthica* seeds were sterilized with 1% sodium hypochlorite solution containing Tween-20 for 5 min and washed five times with sterile water. The seeds were then suspended in 1% agar solution and loaded on 5-mm glass fiber filter paper disks (20–60 seeds/disk). After incubation at 30 °C for 7 days, each disk was transferred into a well of a 48 or 96 well plate. Each well was added with 20 µL of the extract of hydroponic culture media dissolved in 500 µL of sterile water. GR24 (0.1 μM) was used as a positive control and sterile water as a negative control. The 48 or 96 well plates were incubated for 1 or 2 days at 30 °C; then, the percentage of germination was recorded. Each experiment was performed in five biological replicates.

## Supplementary Materials

The following are available online. Figure S1: Three groups of chemicals known as Strigolactone biosynthesis inhibitor, Figure S2: Structure of tested compounds used for screening, Figure S3: Effect of triflumizole on *Striga* germination rate, Figure S4: Pictures of *Striga* gemination.
