# Supplementary material for: Triflumizole as a Novel Lead Compound for Strigolactone Biosynthesis Inhibitor
Source: Molecules. 2020 Nov 25;25(23):5525. doi: 10.3390/molecules25235525 (PMC7728069; doi:10.3390/molecules25235525)
Supplement: Supplementary file 1 [file molecules-25-05525-s001.pdf]

Supplementary Materials: Triflumizole as a Novel Lead Compound for Strigolactone Biosynthesis Inhibitor

Kojiro Kawada [1], Yuya Uchida [1], Ikuo Takahashi [2], Takahito Nomura [3], Yasuyuki Sasaki [1], Tadao Asami[2], Shunsuke Yajima [1] and Shinsaku Ito [1,*]

[1] Department of Bioscience, Tokyo University of Agriculture, Setagaya, Tokyo 156-8502, Japan
[2] Graduate School of Agricultural and Life Sciences, The University of Tokyo, Bunkyo, Tokyo 113-8657, Japan
[3] Center for Bioscience Research and Education, Utsunomiya University, Utsunomiya, Tochigi 321-8505, Japan
* Correspondence: s4ito@nodai.ac.jp; Tel.: +81-3-5477-2365

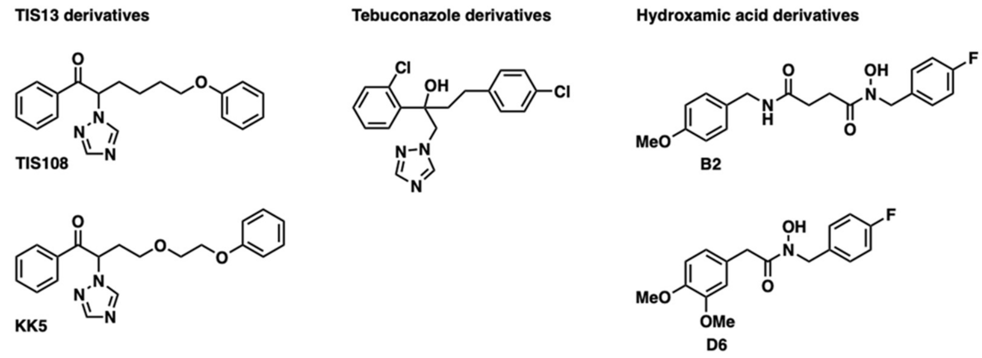

Figure S1. Three groups of chemicals known as Strigolactone biosynthesis inhibitor.

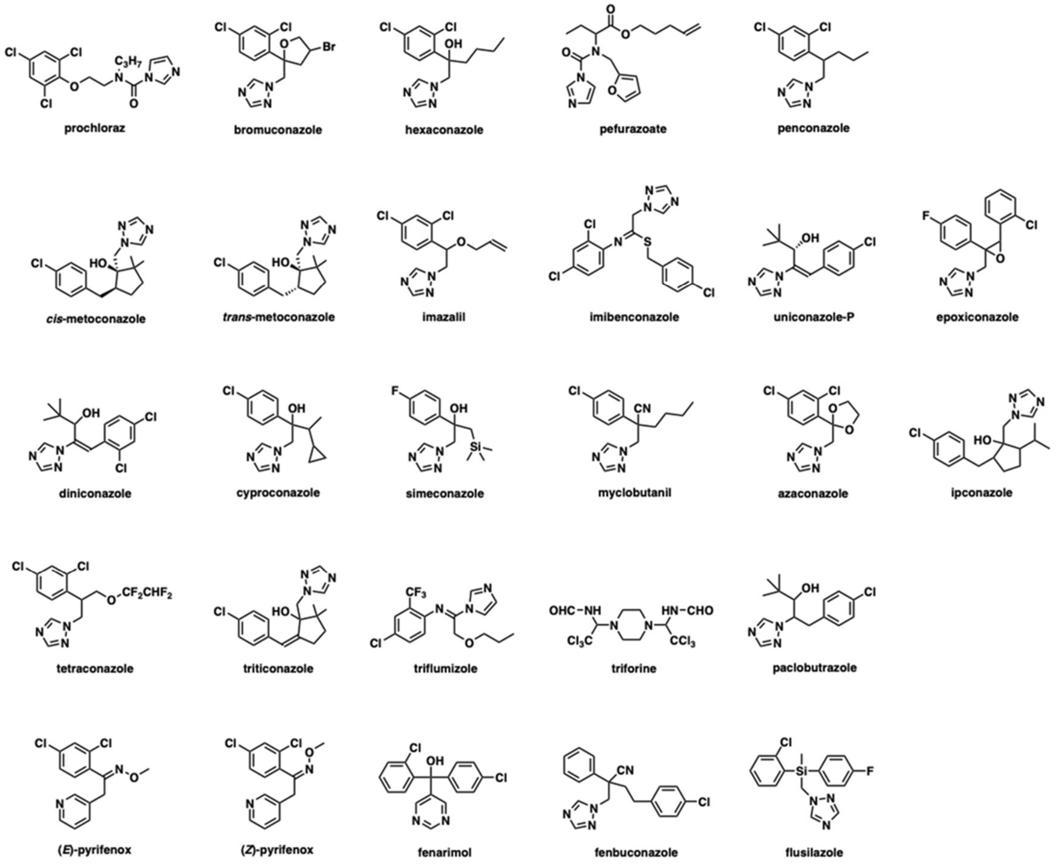

Figure S2. Structure of tested compounds used for screening.

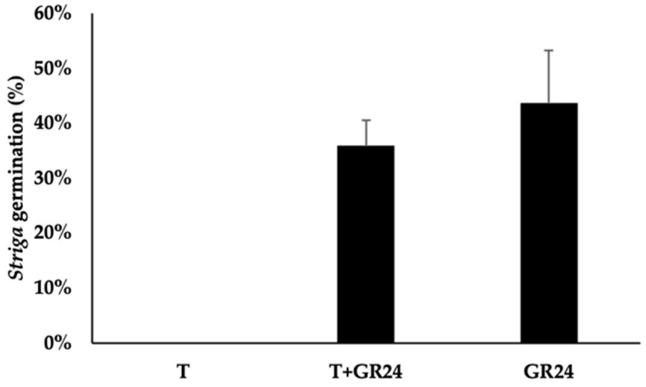

Figure S3. Effect of triflumizole on *Striga* germination rate. In the absence of plants, we extracted hydroponic culture media containing 10 μM triflumizole with ethyl acetate twice. T and T+GR24 means triflumizole-containing extract and the triflumizole-containing extract and 0.1 μM GR24, respectively.

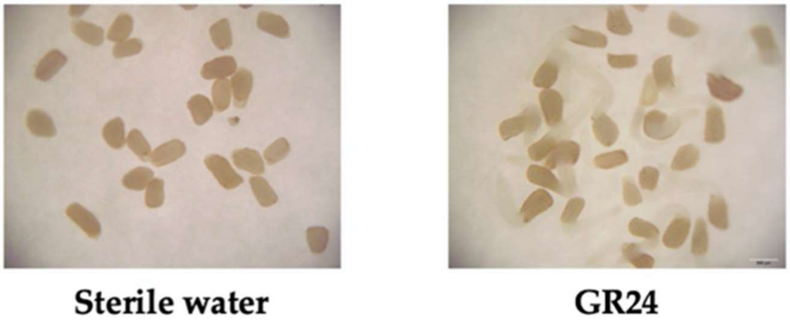

Figure S4. *Striga* seeds.
